# Bayesian Estimation of Multicomponent Stress–Strength Model Using Progressively Censored Data from the Inverse Rayleigh Distribution

**DOI:** 10.3390/e27111095

**Published:** 2025-10-23

**Authors:** Asuman Yılmaz

**Affiliations:** Department of Econometrics, Faculty of Economics and Administrative Sciences, Van Yüzüncü Yıl University, 65080 Van, Turkey; asumanduva@yyu.edu.tr

**Keywords:** multicomponent stress-strength model, inverse Rayleigh distribution, maximum likelihood estimation, Bayesian estimation methods, Monte Carlo simulation

## Abstract

This paper presents a comprehensive study on the estimation of multicomponent stress–strength reliability under progressively censored data, assuming the inverse Rayleigh distribution. Both maximum likelihood estimation and Bayesian estimation methods are considered. The loss function and prior distribution play crucial roles in Bayesian inference. Therefore, Bayes estimators of the unknown model parameters are obtained under symmetric (squared error loss function) and asymmetric (linear exponential and general entropy) loss functions using gamma priors. Lindley and MCMC approximation methods are used for Bayesian calculations. Additionally, asymptotic confidence intervals based on maximum likelihood estimators and Bayesian credible intervals constructed via Markov Chain Monte Carlo methods are presented. An extensive Monte Carlo simulation study compares the efficiencies of classical and Bayesian estimators, revealing that Bayesian estimators outperform classical ones. Finally, a real-life data example is provided to illustrate the practical applicability of the proposed methods.

## 1. Introduction

The inverse Rayleigh (IR) distribution was first presented by Treyer [1] as a model for examining survival and reliability data. Afterward, Voda [2] developed the IR distribution, which works well for simulating early-life failures. Systems with decreasing failure rates, which are typical in mechanical and electronic applications, are especially appropriate for this purpose. Numerous applications have resulted from its analytical tractability and flexibility in reliability analysis.

The probability density function (PDF) and cumulative distribution function (CDF) of the IR distribution are given by:

(1) $$f\left(x;\theta \right)=\frac{2\theta }{x^{3}}e^{-\left(\frac{\theta }{x^{2}}\right)}\;\;\;\;\;\;\;\;\;\;\theta ,\;x>0$$

(2) $$F\left(x;\theta \right)=e^{-\left(\frac{\theta }{x^{2}}\right)}\;\;\;\;\;\theta ,x>0$$

respectively. Here θ is the scale parameter. The shorthand $X\sim IR\left(\theta \right)$ is used to indicate that the random variable X has θ>0. In the field of reliability theory, multicomponent stress–strength reliability (MSSR) models are widely used in reliability systems containing two or more components. A MSSR contains one stress component and k independent strength components. The system remains operational as long as the applied stress does not exceed the strength of at least s−out−of−k components. This framework is particularly applicable in real-world scenarios, such as mechanical, civil, military, and electronic systems, where the partial failure of some components does not necessarily result in total system failure. For example, a bridge supported by k suspension cables can be modeled as an s−out−of−k system, where the structure remains operational as long as at least s cables can bear the dynamic stresses from traffic, wind, or environmental loading. Similarly, in automotive engineering, an engine with eight cylinders may require a minimum of four active cylinders to continue functioning, representing a 4-out-of-8 system.

In such systems, the strength variables $\left(X_{1},\;X_{2},\ldots X_{k}\right)$ are assumed to be independent and identically distributed with CDF $F_{x}\left(x\right)$, while the stress variable independent of the strength has CDF $F_{y}\left(y\right).$ The MSSR model was developed by [3] and described as:

(3) $$R_{s,k}=P\left[at\;least\;of\;the\;X_{1},\;X_{2},\ldots ,X_{k}\;exceed\;Y\;\right]\;R_{s,k}=\sum_{p=s}^{k}\left(\begin{array}{c} k\\ p \end{array}\right)\int_{-\infty }^{+\infty }{\left[1-F_{x}\left(y\right)\right]}^{p}{\left[F_{x}\left(y\right)\right]}^{k-p}dF_{Y}\left(y\right).$$

In recent years, MSSR models have been extensively studied under various probability distributions, including the Weibull distribution [4], generalized inverted exponential distribution [5], and the Marshall–Olkin bivariate Weibull distribution [6]. Moreover, several censoring schemes have been considered to address incomplete data scenarios; see [7,8,9]. Most inferences related to multicomponent reliability have been derived under the assumption of complete data. However, when censoring takes place, inference approaches have received little attention. The ability to effectively and precisely estimate unknown parameters using censored data is important since such data are frequently found, particularly in live studies. The literature has a variety of censoring types that are frequently applied in a wide range of contexts. In this regard, parametric distribution estimate under various censoring techniques has been the subject of numerous investigations. For example [10,11,12,13,14,15,16,17] highlight the practical importance of these methods. Among these censoring types, progressive Type-II censoring has become one of the most preferred methods in recent years because it offers advantages in terms of optimizing experiment duration, reducing costs, and compensating for information loss; see [18].

A progressive censoring scheme can be summarized as follows:

Suppose N identical units are placed in a life test, and from these, a sample size n $\left(n<N\right)$ is observed. At the time of the first failure, a fixed number $R_{1}$ of surviving units are randomly removed from the test. Similarly, at the second failure time, $R_{2}\;$surviving units are withdrawn at random from the remaining units after accounting for prior removals and failures. This procedure continues such that at the nth failure, all remaining surviving units, $R_{n}=N-n-\sum_{i=1}^{n-1}R_{i}.$ See [19] for more details on the progressive censoring scheme.

Nevertheless, there has not been much study carried out on the application of progressive Type-II censoring under the inverse Rayleigh distribution. The inverse Rayleigh distribution is widely used in many studies, especially in reliability theory. In this study, considering all these situations, progressive Type-II censoring under the inverse Rayleigh distribution within the MSSR model is discussed. To our knowledge, no previous comprehensive study has been conducted on this subject. This gap forms the primary motivation for this study. We propose various estimation methods, including maximum likelihood estimation (MLE) and Bayesian inference under different loss functions. The inverse Rayleigh distribution is particularly important in this context because it effectively models failure behaviors that standard monotone failure rate models fail to capture, especially the early-failure period followed by a stable operational phase, which is common in fields such as electronic and mechanical engineering.

The rest of this paper is organized as follows: Section 2 presents the model description and the calculation of MRSS reliability based on the IR distribution. In Section 3.1, maximum likelihood estimators (MLEs) and their asymptotic confidence intervals (ACIs) are derived. Section 3.2 focuses on Bayesian estimation using the squared error loss function (SELF), the Linear exponential (LINEX) loss function, and the generalized exponential loss function (GELF). Bayesian estimators are obtained via the Markov Chain Monte Carlo (MCMC) and Lindley approximation methods. Corresponding Bayesian credible intervals (BCI) for MSSR reliability $R_{s,k}$ are also constructed in this section. A Monte Carlo simulation study to evaluate the performance of the estimators is provided in Section 4. In Section 5, a real life data set is used to illustrate the computations of the MLE and Bayes estimators. Finally, concluding remarks and potential future research directions are discussed in Section 6.

## 2. Model Description

Let $\left(X_{1},X_{2},\ldots ,X_{k}\right)$ be independent strength variables drawn from $IR\left(\theta \right)$ and let Y be the stress variable independently drawn from $IR\left(\alpha \right).$

We assume an s−out−of−k system structure, where the system performs successfully if at least *s* out of *k* components have strengths greater than the applied stress. Thus, by substituting the CDF and PDF of the IR distribution given in Equations (1) and (2) into the Equation (3), the multicomponent reliability $R_{s,k}$ is obtained as follows:

$$R_{s,k}=\sum_{p=s}^{k}\left(\begin{array}{c} k\\ p \end{array}\right){\int_{-\infty }^{+\infty }\left(1-e^{-\left(\frac{\theta }{x^{2}}\right)}\right)}^{p}{\left(e^{-\left(\frac{\theta }{x^{2}}\right)}\right)}^{k-p}\frac{2\alpha }{x^{3}}e^{-\left(\frac{\alpha }{x^{2}}\right)}dx$$

Using the substitution $u=e^{-\left(\frac{\theta }{x^{2}}\right)}$ and simplifying, this integral reduces to a form involving the Beta function:

$$R_{s,k}=\frac{\alpha }{\theta }\int_{0}^{1}{\left(1-u\right)}^{p}u^{k-p+\frac{\alpha }{\theta }-1}=\frac{\alpha }{\theta }B\left(k-p+\frac{\alpha }{\theta },p+1\right).$$

It is even more easily reduced to a closed-form expression:

(4) $$R_{s,k}=\frac{\alpha }{\theta }\sum_{k=s}^{p}\frac{k!}{\left(k-p\right)!}{\left(\prod_{j=0}^{i}\left(k+\frac{\alpha }{\theta }-j\right)\right)}^{-1}.$$

## 3. Parameter Estimation

In this section, parameter estimation is briefly presented using both maximum likelihood estimation and Bayesian estimation methods.

### 3.1. Maximum Likelihood Estimation

We first compute MLEs of parameters θ and α under progressive Type-II censoring. Consider N identical systems subjected to a life testing experiment, each having K components, and n system, each with k components are observed under a progressive Type-II censoring scheme. The observed sample $\left(X_{i1},X_{i2},\ldots ,X_{ik}\right),\;\;\;i=1,2,\ldots ,n$ is obtained from $IR\left(\theta \right)$ using the censoring scheme $\left\{K,k,R_{1},\ldots ,R_{k}\right\}$ Similarly, an independent progressively censored sample $\left\{Y_{1},Y_{2},\ldots ,Y_{n}\right\}$ is observed from an independent $IR\left(\alpha \right)$ with respect to the censoring scheme $\left\{N,n,S_{1},\ldots S_{n}\right\}$. Based on these samples and censoring schemes, the likelihood function for parameters θ and α is obtained as:

(5) $$L\left(\theta ,\alpha \right)=c_{1}\prod_{i=1}^{n}\left(c_{2}\prod_{j=1}^{k}f\left(x_{ij}\right){\left[1-F\left(x_{ij}\right)\right]}^{R_{j}}\right)f\left(y_{i}\right){\left[1-F\left(y_{i}\right)\right]}^{S_{i}}$$

$$c_{1}=N\left(N-S_{1}-1\right)\ldots \left(N-S_{1}-\cdots -S_{n-1}-n+1\right),$$

and

$$c_{2}=K\left(K-R_{1}-1\right)\ldots \left(K-R_{1}-\cdots -R_{k-1}-k+1\right).$$

Using the observed data, the likelihood function can be expressed as follows:

(6) $$L=c_{1}c_{2}^{n}{\left(2\theta \right)}^{nk}{\left(2\alpha \right)}^{k}\prod_{i=1}^{n}\prod_{j=1}^{k}\left(x_{ij}^{-3}e^{-\left(\frac{\theta }{x_{ij}^{2}}\right)}\right){\left(1-x_{ij}^{-3}e^{-\left(\frac{\theta }{x_{ij}^{2}}\right)}\right)}^{R_{j}}\prod_{i=1}^{n}\left(y_{i}^{-3}e^{-\left(\frac{\alpha }{y_{i}^{2}}\right)}\right){\left(1-y_{i}^{-3}e^{-\left(\frac{\alpha }{y_{i}^{2}}\right)}\right)}^{S_{i}}.$$

Then, the log-likelihood function is:

(7) $$logL=log\left(c_{1}\right)+nlog\left(c_{2}\right)+2nklog\left(\theta \right)+2klog\left(\alpha \right)\;-3\sum_{i=1}^{n}\left({logx}_{ij}+logy_{i}\right)-\sum_{i=1}^{n}\left(\sum_{j=1}^{k}\theta x_{ij}^{-2}+\alpha y_{i}^{-2}\right)+\sum_{i=1}^{n}\sum_{j=1}^{k}R_{j}log\left(w\right)+\sum_{i=1}^{n}S_{i}\log\left(v\right).$$

Here, w and v are defined as $w=\left(1-{x_{ij}^{-3}e}^{-\left(\theta /x_{ij}^{2}\right)}\right)$ and $y=\left(1-y_{i}^{-3}e^{-\left(\alpha /y_{i}^{2}\right)}\right)$.

By taking the derivatives of (7) with respect to θ and α the likelihood equations are derived in the following forms:

(8) $$\frac{\partial lnL}{\partial \theta }=\frac{nk}{\theta }-\sum_{i=1}^{n}\sum_{j=1}^{k}\frac{1}{x_{ij}^{2}}+\sum_{i=1}^{n}\sum_{j=1}^{k}R_{j}\frac{{x_{ij}^{-3}e}^{-\left(\theta /x_{ij}^{2}\right)}}{x_{ij}^{2}\left(1-{x_{ij}^{-3}e}^{-\left(\theta /x_{ij}^{2}\right)}\right)}=0,$$

and

(9) $$\frac{\partial lnL}{\partial \alpha }=\frac{k}{\alpha }-\sum_{i=1}^{n}\frac{1}{y_{i}^{2}}+\sum_{i=1}^{n}S_{i}\frac{y_{i}^{-3}e^{-\left(\alpha /y_{i}^{2}\right)}}{y_{i}^{2}\left(1-y_{i}^{-3}e^{-\left(\alpha /y_{i}^{2}\right)}\right)}=0.$$

Let us define the MLEs of θ and α, as ${\hat{\theta }}_{MLE}$ and ${\hat{\alpha }}_{MLE}$, respectively. However, the ${\hat{\theta }}_{MLE}$ and ${\hat{\alpha }}_{MLE}$ can not be obtained explicitly by solving Equations (8) and (9). Numerical methods should be used to solve these equations. Therefore, the ${\hat{\theta }}_{MLE}$ and ${\hat{\alpha }}_{MLE}$ are obtained by using the well-known Newton–Raphson iteration in this study. Therefore, MLE of $R_{s,k}$ can be obtained as:

(10) $${\hat{R}}_{s,k}=\frac{\hat{\alpha }}{\hat{\theta }}\sum_{k=s}^{p}\frac{k!}{\left(k-p\right)!}{\left(\prod_{j=0}^{i}\left(k+\frac{\hat{\alpha }}{\hat{\theta }}-j\right)\right)}^{-1}.$$

In this subsection, the ACI for $R_{s,k}$ is obtained by using the fact that the distribution of MLE is asymptotically normal. Let$\;V=\left(\theta ,\;\alpha \right)$; the observed Fisher information matrix is denoted by $I\left(V\right)$ and is given by:

(11) $$I\left(V\right)=\left[I_{ij}\right]=\left[\frac{-\partial^{2}V}{\partial V_{i}\partial V_{j}}\right]={\left[\frac{-\partial^{2}l}{\partial V_{i}\partial V_{j}}\right]}_{V=\hat{V}},i,j=1,2\;$$

where

(12) $$I_{11}=\frac{-nk}{\theta^{2}}-\sum_{i=1}^{n}\sum_{j=1}^{k}R_{j}\frac{{x_{ij}^{-3}e}^{-\left(\theta /x_{ij}^{2}\right)}}{{\left(x_{ij}^{2}\left(1-x_{ij}^{-3}e^{-\left(\theta /x_{ij}^{2}\right)}\right)\right)}^{2}},$$

(13) $$I_{22}=-\frac{k}{\alpha^{2}}-\sum_{i=1}^{n}S_{i}\frac{y_{i}^{-3}e^{-\left(\alpha /y_{i}^{2}\right)}}{{\left(y_{i}^{2}\left(1-y_{i}^{-3}e^{-\left(\alpha /y_{i}^{2}\right)}\right)\right)}^{2}},$$

and

$$I_{12}=I_{21}=0.$$

Then the asymptotic variance of ${\hat{R}}_{s,k}$ is obtained as:

(14) $$Var\left({\hat{R}}_{s,k}\right)={\left(\frac{\partial R_{s,k}}{\partial \theta }\right)}^{2}I_{11}^{-1\;}+{\left(\frac{\partial R_{s,k}}{\partial \alpha }\right)}^{2}I_{22}^{-1}$$

To avoid the complexity involved in the derivation of${\;R}_{s,k}$, we consider only the cases $\left(s,k\right)=\left(1,3\right)$ and,$\;\left(2,4\right)$ for which the expressions and their derivatives are obtained separately and presented below. Similar procedures have also been carried out under different scenarios by [9,20].

(15) $$R_{1,3}=\frac{3\theta }{3\theta +\alpha },\frac{{\partial R}_{1,3}}{\partial \theta }=\frac{3\alpha }{{\left(3\theta +\alpha \right)}^{2}}\;\mathrm{and}\;\frac{{\partial R}_{1,3}}{\partial \alpha }=-\frac{3\alpha }{{\left(3\theta +\alpha \right)}^{2}}$$

(16) $$R_{2,4}=\frac{12\theta^{2}}{\left(4\theta +\alpha \right)\left(3\theta +\alpha \right)},\frac{{\partial R}_{2,4}}{\partial \theta }=\frac{12\alpha \theta \left(7\theta +2\alpha \right)}{{\left[\left(4\theta +\alpha \right)\left(3\theta +\alpha \right)\right]}^{2}},\mathrm{and}\;\frac{{\partial R}_{2,4}}{\partial \alpha }=-\frac{12\theta^{2}\left(7\theta +2\alpha \right)}{{\left[\left(4\theta +\alpha \right)\left(3\theta +\alpha \right)\right]}^{2}}.$$

Note that all parameters are evaluated at MLEs $\left(\theta ,\alpha \right)$ Thus, the $100\left(1-\gamma \right)\%$ ACI of $R_{s,k}$ is given by:

(17) $$\left(R_{s,k}-Z_{\gamma /2}\sqrt{Var\left({\hat{R}}_{s,k}\right)}\;\;\;,R_{s,k}+Z_{\gamma /2}\sqrt{Var\left({\hat{R}}_{s,k}\right)}\right)$$

where $Z_{\gamma }$ is $100\left(1-\gamma \right)-th$ percentile of $N\left(0,1\right).$

### 3.2. Bayesian Inference

This section presents a Bayesian approximation for estimating the system reliability $R_{s,k}$ of the MSSR model under progressively Type-II censored data, considering both symmetric and asymmetric loss functions. Furthermore, MCMC and Lindley approximations are considered to compute the Bayesian estimators. In Bayesian inference, the selection of an appropriate loss function is important because it affects the parameter estimation. The SELF is widely used due to its symmetric structure, as it assigns equal weights to both overestimation and underestimation. It is also commonly preferred in practice because it involves simple computations and does not require intensive numerical procedures, see [21].

SELF is given by as below:

$$L_{SELF}\left(\hat{\theta },\;\theta \right)={\left(\hat{\theta }-\theta \right)}^{2},$$

where $\hat{\theta }$ is the estimate of the parameter θ. The posterior mean is the Bayesian estimate of the parameter θ under SELF, which is given as:

$${\hat{\theta }}_{SELF}=E\left(\theta \right)$$

Numerous studies have indicated that overestimation is more critical than underestimation. In such scenarios, asymmetric loss functions are more appropriate; see [22]. This study uses the GELF and LINEX asymmetric loss functions for Bayesian parameter estimation. These functions effectively represent the asymmetric effect of estimation errors, leading to more reliable results [22,23]. Due to their flexibility and ability to capture asymmetric estimation errors, these functions have been widely applied in diverse statistical modeling contexts, see [24,25].

The mathematical form of the LINEX loss function proposed by [23] is:

(18) $${\hat{L}}_{LINEX}=q\left\{e^{q\left(\hat{\theta }-\theta \right)}-q\left(\hat{\theta }-\theta \right)-1\right\};\;\;\;q\neq 0.$$

The following formula gives the Bayes estimation under this loss function:

(19) $${\hat{\theta }}_{LINEX}=-\frac{1}{q}ln\left(E\left(\left(e^{-q\theta }|x\right)\right)\right)$$

Another asymmetric loss function called a GELF was also proposed by [26] and is as follows:

(20) $${\hat{L}}_{GELF}=q\left\{{\left(\frac{\hat{\theta }}{\theta }\right)}^{q}-q\left(\frac{\hat{\theta }}{\theta }\right)-1\right\};\;\;\;\;q\neq 0$$

Under this loss function, the Bayes estimation is given by the following equation:

(21) $${\hat{\theta }}_{GELF}=E{\left(\theta^{-q}|x\right)}^{\frac{1}{q}}$$

The direction and degree of symmetry are expressed by the sign and magnitude of q in the GELF and LINEX loss functions.

In Bayesian inference, both the choice of loss function and prior distribution significantly influence the resulting estimators. The prior distribution expresses prior knowledge about the unknown parameters. This information contributes to more accurate and reliable parameter estimation. It is crucial to Bayesian inference and is particularly useful when there is not much information available [27]. In this study, independent gamma priors are assigned to the parameters due to their flexibility and suitability. It is commonly used in the literature for modeling shape and scale parameters in lifetime distributions, see [28,29].

Thus, the proposed prior PDFs of the independent and gamma priors for parameters θ and α are given as:

$$\pi_{1}\left(\theta \right)=\frac{b_{1}^{a_{1}}}{\Gamma \left(a_{1}\right)}\theta^{a_{1}-1}e^{-b_{1}\theta },\;\mathrm{and}\;\pi_{2}\left(\alpha \right)=\frac{b_{2}^{a_{2}}}{\Gamma \left(a_{2}\right)}\alpha^{a_{2}-1}e^{-b_{2}\alpha }$$

Here, the hyper-parameters $\left(a_{1},\;a_{2},b_{1},\;b_{2}\right)$ are known and positive. The joint prior distributions for θ and α are:

(22) $${\pi \left(\theta ,\alpha \right)=\pi }_{1}\left(\theta \right)\pi_{2}\left(\alpha \right)=\frac{b_{1}^{a_{1}}b_{2}^{a_{2}}}{\Gamma \left(a_{1}\right)\Gamma \left(a_{2}\right)}\theta^{a_{1}-1}\alpha^{a_{2}-1}.$$

Based on Equations (6) and (22), the joint posterior distribution of the parameters θ and α can be written as follows:

(23) $$\pi \left(\left(\theta ,\alpha |x\right)\right)=\frac{L\left(x|\theta ,\alpha \right)\pi \left(\theta ,\alpha \right)}{\int_{0}^{\infty }\int_{0}^{\infty }L\left(x|\theta ,\alpha \right)\pi \left(\theta ,\alpha \right)d\theta d\alpha }\propto \theta^{nk+a_{1}-1}\alpha^{k+a_{2}-1}e^{-b_{1}\theta -b_{2}\alpha }*\;\prod_{i=1}^{n}\prod_{j=1}^{k}\left(x_{ij}^{-3}e^{-\left(\theta /x_{ij}^{2}\right)}\right){\left(1-x_{ij}^{-3}e^{-\left(\theta /x_{ij}^{2}\right)}\right)}^{R_{j}}\prod_{i=1}^{n}\left(y_{i}^{-3}e^{-\left(\alpha /y_{i}^{2}\right)}\right){\left(1-y_{i}^{-3}e^{-\left(\alpha /y_{i}^{2}\right)}\right)}^{S_{i}}.$$

Then, based on Equation (23), the conditional posterior density functions of parameters θ and α are given as:

(24) $$\pi_{1}\left(\theta ,\alpha |x\right)\propto \theta^{nk+a_{1}-1}e^{-b_{1}\theta }\prod_{i=1}^{n}\prod_{j=1}^{k}\left(x_{ij}^{-3}e^{-\left(\theta /x_{ij}^{2}\right)}\right){\left(1-x_{ij}^{-3}e^{-\left(\theta /x_{ij}^{2}\right)}\right)}^{R_{j}},$$

(25) $$\pi_{2}\left(\theta ,\alpha |x\right)\propto \alpha^{k+a_{2}-1}e^{-b_{2}\alpha }\prod_{i=1}^{n}\left(y_{i}^{-3}e^{-\left(\alpha /y_{i}^{2}\right)}\right){\left(1-y_{i}^{-3}e^{-\left(\alpha /y_{i}^{2}\right)}\right)}^{S_{i}}.$$

To calculate the Bayes estimators of the parameters in Equations (24) and (25), the expected values of the conditional posterior distributions must be evaluated. Since these expected values cannot be computed analytically, this study uses two alternative methods. Lindley’s approximation and MCMC techniques are employed to obtain the Bayes estimators. The following subsection gives a brief discussion of these techniques.

#### 3.2.1. Lindley Approximation

Lindley’s approximation is widely used to obtain Bayesian estimators when analytical solutions are not feasible; see [30]. Under the SELF, the Bayes estimator of ${\hat{R}}_{s,k}$ is given as follows:

(26) $${\hat{R}}_{s,k}={\hat{R}}_{SELF}+{\left[R_{1}d_{i}+R_{2}d_{i}+d_{3}+d_{4}\right]+0.5\left[A\left(R_{1}\sigma_{11}+R_{2}\sigma_{12}\right)+B\left(R_{1}\sigma_{21}+R_{2}\sigma_{22}\right)\right]}_{\hat{\alpha },\hat{\theta }}\;$$

Here

$$d_{i}=\rho_{1}\sigma_{i1}+\rho_{2}\sigma_{i2},\;i=1,2,\;\;d_{3}=R_{12}\rho_{12},d_{4}=0.5\left[R_{11}\sigma_{11}+R_{22}\sigma_{22}\right]$$

$$A=L_{111}\sigma_{11}+2L_{121}\sigma_{12}+L_{221}\sigma_{22},B=L_{112}\sigma_{11}+2L_{122}\sigma_{12}+L_{222}\sigma_{22}.$$

In our scenario, $\left(\theta_{1},\theta_{2}\right)=\left(\theta ,\alpha \right)$ and $u=R\left(\theta ,\alpha \right)=R_{s,k}$, where $R_{s,k}$ and $\left(I_{11},\;I_{22}\right)$ are given in Equations (4), (13), and (14), respectively. Other expressions related to Equation (26) are given below:

$$\rho_{1}=\frac{a_{1}-1}{\theta }-b_{1},\rho_{2}=\frac{a_{2}-1}{\alpha }-b_{2},I_{12}=I_{21}=0,\;\sigma_{ij}=\left(i,j\right)\;th,$$

element in the inverse of matrix $\left[-I_{i,j}\right];i=j=1,2,$

$$L_{111}=\frac{2nk}{\theta^{3}}+\sum_{i=1}^{n}\sum_{j=1}^{k}R_{j}\frac{e^{-\left(\theta /x_{ij}^{2}\right)}\left(1+{x_{ij}^{-3}e}^{-\left(\theta /x_{ij}^{2}\right)}\right)}{{\left(x_{ij}^{3}\left(1-x_{ij}^{-3}e^{-\left(\theta /x_{ij}^{2}\right)}\right)\right)}^{3}},$$

$$L_{222}=\frac{2k}{\alpha^{3}}+\sum_{i=1}^{n}S_{i}\frac{e^{-\left(\alpha /y_{i}^{2}\right)}\left(1+y_{i}^{-3}e^{-\left(\alpha /y_{i}^{2}\right)}\right)}{{\left(y_{i}^{3}\left(1-y_{i}^{-3}e^{-\left(\alpha /y_{i}^{2}\right)}\right)\right)}^{3}},$$

and other $\;L_{ijk}=0$. In our case, $R_{1}=\frac{\partial R_{s,k}}{\partial \theta },\;\;R_{2}=\frac{{\partial R}_{s,k}}{\partial \alpha }$ are given in Equations (15) and (16) for $\left(s,k\right)=\left(1,3\right)$ and $\left(2,4\right)$ respectively. Furthermore,$\;R_{11}=\frac{{\partial^{2}R}_{s,k}}{{\partial \theta }^{2}},\;\;R_{22}=\frac{{\partial^{2}R}_{s,k}}{\partial \alpha^{2}}$ are computed as follows:

$$R_{11}=\frac{{\partial^{2}R}_{1,3}}{{\partial \theta }^{2}}=-\frac{18\alpha }{{\left(3\theta +\alpha \right)}^{3}}\;,\mathrm{and}\;R_{22}=\frac{{\partial^{2}R}_{1,3}}{{\partial \alpha }^{2}}=\frac{6\theta }{{\left(3\theta +\alpha \right)}^{3}},$$

$$R_{11}=\frac{{\partial^{2}R}_{2,4}}{{\partial \theta }^{2}}=\frac{12\alpha \left[\left(14\theta +2\alpha \right)\left(4\theta +\alpha \right)\left(3\theta +\alpha \right)-2\theta \left(\left(7\theta +2\alpha \right)\left(24\theta +7\alpha \right)\right)\right]}{{\left[\left(4\theta +\alpha \right)\left(3\theta +\alpha \right)\right]}^{3}},$$

$$R_{22}=\frac{{\partial^{2}R}_{2,4}}{{\partial \alpha }^{2}}=\frac{24\theta^{2}\left[{\left(7\theta +2\alpha \right)}^{2}-\left(4\theta +\alpha \right)\left(3\theta +\alpha \right)\right]}{{\left[\left(4\theta +\alpha \right)\left(3\theta +\alpha \right)\right]}^{3}}.$$

As mentioned earlier, using Equation (26), the Bayesian estimator of $R_{1,3}$ and $R_{2,4}$ under the SELF is obtained as follows:

$$E\left({\hat{R}}_{1,3}|x\right)=\frac{3\hat{\theta }}{3\hat{\theta }+\hat{\alpha }}+\left[{\hat{R}}_{1}{\hat{d}}_{i}+{\hat{R}}_{2}{\hat{d}}_{i}+{\hat{d}}_{3}+{\hat{d}}_{4}\right]+0.5\left[\hat{A}\left({\hat{R}}_{1}{\hat{\sigma }}_{11}+{\hat{R}}_{2}{\hat{\sigma }}_{12}\right)+\hat{B}\left({\hat{R}}_{1}{\hat{\sigma }}_{21}+{\hat{R}}_{2}{\hat{\sigma }}_{22}\right)\right]$$

and

$$E\left({\hat{R}}_{2,4}|x\right)=\frac{12\theta^{2}}{\left(4\theta +\alpha \right)\left(3\theta +\alpha \right)}+\left[{\hat{R}}_{1}{\hat{d}}_{i}+{\hat{R}}_{2}{\hat{d}}_{i}+{\hat{d}}_{3}+{\hat{d}}_{4}\right]+0.5\left[\hat{A}\left({\hat{R}}_{1}{\hat{\sigma }}_{11}+{\hat{R}}_{2}{\hat{\sigma }}_{12}\right)+\hat{B}\left({\hat{R}}_{1}{\hat{\sigma }}_{21}+{\hat{R}}_{2}{\hat{\sigma }}_{22}\right)\right].$$

Now, the Bayesian estimator of $u=R\left(\theta ,\alpha \right)={\left(e^{R_{s,k}}\right)}^{-q}$ is computed under the LINEX loss function. In the case of $\left(s,k\right)=\left(1,3\right)$ is presented in detail as follows:

$$R_{1,3}=e^{-q\left(\frac{3\theta }{3\hat{\theta }+\alpha }\right)},{\;R}_{1}=\frac{\partial R_{1,3}}{\partial \theta }={-\frac{3\alpha q}{{\left(3\theta +\alpha \right)}^{2}}e}^{-q\left(\frac{3\theta }{3\theta +\alpha }\right)}$$

$$R_{11}=\frac{\partial^{2}R_{1,3}}{\partial \theta^{2}}={\frac{9\alpha q\left(2\alpha +6\theta +\alpha q\right)}{{\left(3\theta +\alpha \right)}^{4}}e}^{-q\left(\frac{3\theta }{3\theta +\alpha }\right)}$$

$$R_{2}=\frac{\partial R_{1,3}}{\partial \alpha }=\frac{3\theta q}{{\left(3\theta +\alpha \right)}^{2}}e^{-q\left(\frac{3\theta }{3\theta +\alpha }\right)},\;R_{2}=\frac{{\partial^{2}R}_{1,3}}{\partial \alpha^{2}}=\frac{-3\theta q\left(2\alpha +6\theta -3q\theta \right)}{{\left(3\theta +\alpha \right)}^{4}}e^{-q\left(\frac{3\theta }{3\theta +\alpha }\right)},\;R_{ij}=0,\;i\neq j,\;i,j=1,2,$$

respectively.

The Bayes estimator of $R_{1,3}$ under the LINEX loss function using the Lindley approximation is obtained as:

$$E\left(e^{-q\left({\hat{R}}_{1,3}\right)}|x\right)=\frac{1}{q}ln\left(\left(e^{{\left({\hat{R}}_{1,3}\right)}^{-q}}\right)+\left[{\hat{R}}_{1}{\hat{d}}_{i}+{\hat{R}}_{2}{\hat{d}}_{i}+{\hat{d}}_{3}+{\hat{d}}_{4}\right]+0.5\left[\hat{A}\left({\hat{R}}_{1}{\hat{\sigma }}_{11}+{\hat{R}}_{2}{\hat{\sigma }}_{12}\right)+\hat{B}\left({\hat{R}}_{1}{\hat{\sigma }}_{21}+{\hat{R}}_{2}{\hat{\sigma }}_{22}\right)\right]\right)$$

Lastly, the Bayesian estimator of $u=R\left(\theta ,\alpha \right)=R_{1,3}^{-q}$ is under the GELF given as follows:

$${\;R}_{1}=\frac{\partial R_{1,3}}{\partial \theta }=\frac{3q\alpha }{{\left(3\theta +\alpha \right)}^{2}}{\left(\frac{3\theta }{3\theta +\alpha }\right)}^{-q-1},\;{\;R}_{11}=\frac{\partial^{2}R_{1,3}}{\partial \theta^{2}}=\frac{9q\alpha \left(-6\theta +\alpha \left(q-1\right)\right)}{{\left(3\theta +\alpha \right)}^{4}}{\left(\frac{3\theta }{3\theta +\alpha }\right)}^{-q-2},$$

$${\;R}_{2}=\frac{\partial R_{1,3}}{\partial \alpha }=\frac{-3q\theta }{{\left(3\theta +\alpha \right)}^{2}}{\left(\frac{3\theta }{3\theta +\alpha }\right)}^{-q-1},\;{\;R}_{22}=\frac{\partial^{2}R_{1,3}}{\partial \alpha^{2}}=\frac{9q\theta^{2}\left(q-7\right)}{{\left(3\theta +\alpha \right)}^{4}}{\left(\frac{3\theta }{3\theta +\alpha }\right)}^{-q-2}$$

$$R_{ij}=0,\;i\neq j,\;i,j=1,2,$$

and we have

$$E\left({\hat{R}}_{1,3}^{-q}|x\right)={\left(\left(e^{{\left({\hat{R}}_{1,3}\right)}^{-q}}\right)+\left[{\hat{R}}_{1}{\hat{d}}_{i}+{\hat{R}}_{2}{\hat{d}}_{i}+{\hat{d}}_{3}+{\hat{d}}_{4}\right]+0.5\left[\hat{A}\left({\hat{R}}_{1}{\hat{\sigma }}_{11}+{\hat{R}}_{2}{\hat{\sigma }}_{12}\right)+\hat{B}\left({\hat{R}}_{1}{\hat{\sigma }}_{21}+{\hat{R}}_{2}{\hat{\sigma }}_{22}\right)\right]\right)}^{-1/q}.$$

Using the Lindley approximation method, the Bayesian estimate for $\left(s,k\right)=\left(2,4\right)$ can be calculated similarly under the LINEX loss function and GELF.

#### 3.2.2. Gibbs Sampling

We now apply the Gibbs sampling technique, a subclass of Monte Carlo Markov Chain (MCMC) method, to derive the Bayesian estimate of $R_{s,k}$. The conditional posterior distributions in Equations (24) and (25) do not have closed-form expressions. The posterior densities of θ and α are similar to normal distributions. Consequently, random samples can be efficiently generated using the Metropolis–Hastings (MH) algorithm. Metropolis et al. [31] first presented this algorithm, which uses a normal proposal distribution.

The steps of Gibbs sampling are described as follows:

Step 1: Start with an initial guess $\left(\theta^{0},\;\alpha^{0}\right)=\left(\hat{\theta },\;\hat{\alpha }\right).$

Step 2: Set j=1.

Step 3: Using the M–H algorithm, generate a posterior sample for $\theta^{j}$ and $\alpha^{j}$ from Equations (24) and (25) with normal proposal distributions $N\left(\theta^{j-1},\;Var\left(\theta \right)\right)$ and $N\left(\alpha^{j-1},\;Var\left(\alpha \right)\right),$ respectively.

i. Generate proposal $\theta^{*}$ from $N\left(\theta^{j-1},\;Var\left(\theta \right)\right)$ and $\alpha^{*}$ from $N\left(\alpha^{j-1},\;Var\left(\alpha \right)\right).$

ii. Calculate the acceptance probability

$p_{\theta }=min\left[1,\frac{\pi_{\theta }\left(\theta^{*}|,\;x\right)}{\pi_{\theta }\left(\theta^{j-1}|\alpha^{j-1},\;x\right)}\right]$ and $p_{\alpha }=min\left[1,\frac{\pi_{\alpha }\left(\alpha^{*}|\theta_{j},\;x\right)}{\pi_{\theta }\left(\alpha^{j-1}|\theta^{j},\;x\right)}\right]$

Step 4. Generate a random $u_{1}$ and $u_{2}$ from the uniform $\left(0,1\right)$ distribution.

Step 5. If, $p_{\theta }\geq u_{1},$ $\theta^{j}=\theta^{*}$ otherwise$\;\theta^{j}=\theta^{j-1}$,

If $p_{\alpha }\geq u_{2},$ $\alpha^{j}=\alpha^{*}$ otherwise $\alpha^{j}=\alpha^{j-1}.$

Step 6. Compute $R_{s,k}^{j}$ using $\theta^{j}$ and $\alpha^{j}.$

Step 7. Set j=j+1.

Step 8. Repeat 3–7 N times, and obtain the posterior sample${\;R}_{s,k}^{j},\;\;j=1,\ldots ,N.$

Step 9. The Bayes estimators of $R_{s,k\;}$ using MCMC under the SELF, LINEX, and GELF are given as follows:

$${\hat{R}}_{s,k,SELF}=\frac{1}{N}\left(\sum_{j=M+1}^{N}R_{s,k}^{j}\right),\;{\hat{R}}_{s,k,LINEX}=\frac{-1}{q}ln\left(\frac{1}{N}\sum_{j=M+1}^{N}R_{s,k}^{j}\right),\mathrm{and}$$

$${\hat{R}}_{s,k,GELF}={\left(\frac{1}{N}\sum_{j=M+1}^{N}{\left(R_{s,k}^{j}\right)}^{-q}\right)}^{\frac{-1}{q}}.$$

respectively. Here, M is the burn-in period. We construct the $100\left(1-\gamma \right)\%$ the Bayesian credible intervals of the MRSS using Chen and Shao [32].

## 4. Simulation Study

In this section, an effective Monte Carlo simulation study is conducted to evaluate the performance the of MLE and Bayesian estimations of the $R_{s,k}$. In the Bayes case, two different priors were used. For the first case,${\;a}_{1}=a_{2}=b_{1}=b_{2}=0$ were taken and called them Prior-I (non-informative prior). For the second case, the true values of the parameters were selected as the prior mean. In other words, $\alpha =a_{1}/b_{1}$ and$\;\theta =a_{2}/b_{2}$ are taken and them called Prior-II (informative prior). The real values of model parameters are generally unknown in practical applications. However, in this study, the informative priors were determined using true parameter values within a simulated scenario. This approach allows us to evaluate the impact of informative priors on estimation accuracy under ideal conditions. In real-world applications, such priors can be derived from sources such as historical data or expert opinions, see [33].

The Bayes estimators of the $R_{s,k}$ under SELF, LINEX, and GELF with these priors were found using the Lindley and MCMC approximations. This study compares interval and point estimations. The absolute bias (Abias) and mean square error (MSE) criteria were used to evaluate the point estimations. The average confidence lengths (ACI), the lengths (BCI), and the coverage probability (CP) were used to evaluate the interval estimations. The simulation results are based on 5000 replications and all the computations are performed in Matlab R2013.

The MSSR data can be generated using the following procedure.

Here, K denotes the total number of components in a system, of which k failures are observed and the remaining $\left(K-k\right)$ are censored. Similarly, N is the total number of systems under test, and only n system failures are observed, with the remaining $\left(N-n\right)$ censored. Uniform $U\left(0,1\right)$ samples are generated under the schemes$\;\left(K,,R_{1},\ldots R_{k}\right)$ for strength and $\left(N,n,\;S_{1},\ldots S_{n}\right)\;$for stress, then transformed into the desired distributions via the inverse transformation method. For more detailed information on this algorithm, please refer to [34].

These schemes represent the progressive censoring plans applied to the strength and stress variables. Lifetime experiments are conducted on N systems, each consisting of K components, and data are collected from n systems with k observed failures using the specified censoring schemes (C.S). Note that the sort like notation $\left(0,1\right)*4$ is used for $\left(0,1,0,1,0,1,0,1\right).$ Different censoring schemes are given in Table 1.

We consider two sets of arbitrarily selected parameter values for comparing the various estimators of $R_{s,k}:$ $\theta_{1}=\left(\theta ,\alpha \right)=\left(1.5,\;\;1.2\right)$ and $\theta_{2}=\left(0.5,\;2\right)$. Since the simulation results are similar, the results in Table 2 are summarized only for $\theta_{1}=\left(\theta ,\alpha \right)=\left(1.5,\;\;1.2\right)$.

**Figure 1:**
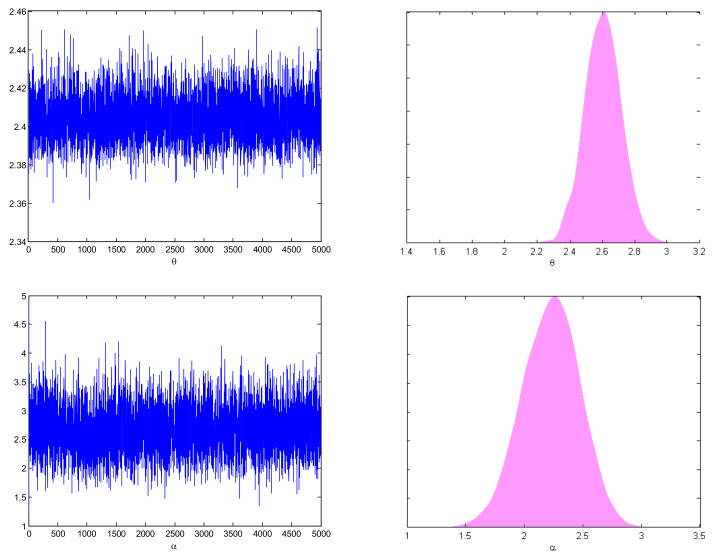
Trace and posterior density plots of θ and α using $\left(R_{3},\;\;S_{6}\right)$.

**Figure 2:**
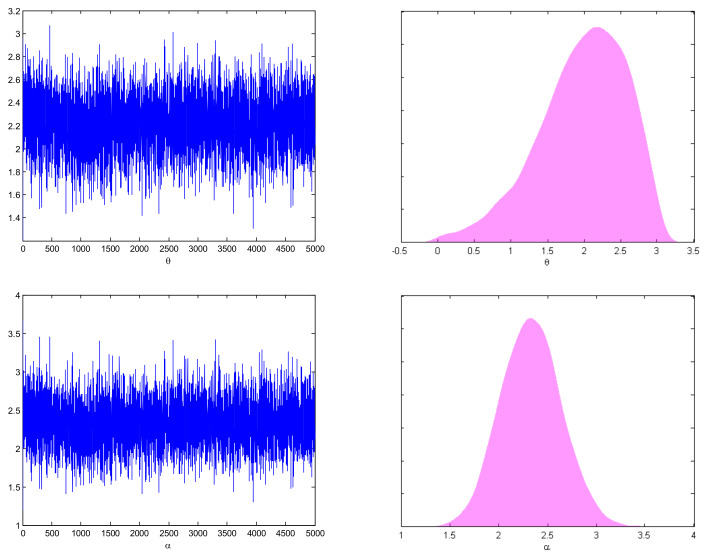
Trace and posterior density plots of θ and α using $\left(R_{2},\;\;S_{5}\right)$.

**Figure 3:**
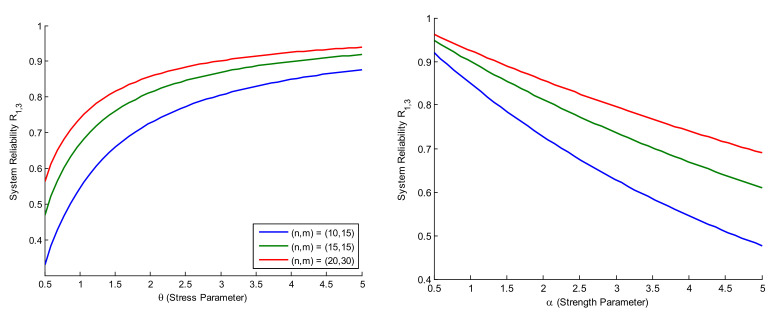
Reliability curves under θ and α parameters with different sample sizes.

Table 3 reports ACI, BCI, and CP values under the defined estimation methods. Specifically, ACI corresponds to MLE, while BCI refers to the interval widths obtained via MCMC estimation under all the proposed loss functions. Furthermore, Figure 1 and Figure 2 provide trace plots and posterior density plots under Prior-I for two different censoring schemes, illustrating the convergence and distributional characteristics of the Bayesian estimators. Furthermore, reliability curves for different sample sizes $\left(n,m\right)$ and parameter values α and θ were generated for both $R_{1,3}$ and $R_{2,4}$. However, since the results are similar, only the reliability curves for $R_{1,3}$ are given in Figure 3 for brevity. Additionally, Gelman and Rubin’s convergence diagnostic method was computed to check the Markov chain. In order to observe the convergence of the MCMC results, Gelman and Rubin [35] developed an indicator known as the potential scale reduction factor to determine whether intra-chain and inter chain variances difference significantly. The produced Markov chains are probably going to have converged to a single target distribution if the potential scale reduction factor value is near 1. For this, we constructed posterior samples with three different initial values, and we determined the value of the potential scale reduction factor, which comes out to be 1. Details can be found in [36]. The MCMC (Markov Chain Monte Carlo) method was finally ran for 5000 iterations, discarding the first 1000 as burn-in, in order to show convergence.

As shown in Table 2, Bayesian methods perform better than the MLE method in terms of Abias and MSE values. When Prior-I and Prior-II are compared, it is observed that Prior-II has smaller Abias and MSE values in most cases. Similarly, when the loss functions are compared to each other, asymmetric loss functions outperform SELF under both Prior-I and Prior-II.

Furthermore, estimation performance varies across censoring schemes. In general, schemes involving later removals lead to more accurate estimates, while early removals result in relatively higher Abias and MSE criteria. 

As shown in Table 3 BCIs constructed using Prior-II are generally shorter than those under Prior-I, while still maintaining coverage probabilities close to the nominal 95% level. Moreover, BCIs under both priors perform better than ACIs in terms of coverage accuracy and interval precision. That is, as can be seen in Table 3, the estimated results are very close to the true values, which shows that the proposed method performs well.

Figure 1 and Figure 2 present the trace and posterior density plots of the parameters θ and α under Prior-I for the cases $\left(R_{3}-S_{6}\right)$ and $\left(R_{2}-S_{5}\right),\;$indicating good mixing and convergence for both priors. Figure 3 reveals that $R_{1,3}$ increases with increasing θ (stress parameter) and decreases with increasing α (strength parameter). Moreover, more reliable system estimates are usually generated by different sample sizes $\left(n,m\right).$

## 5. Real Data Set

In this section, we present an application of the proposed methods using a real data set taken from the literature. The dataset, originally reported by [37], was collected to assess the potential occurrence of extreme drought during summer. It is perfect to illustrate the effectiveness of proposed estimating techniques under progressive censoring due to its structure, where matrices X and Y represent for MRSS variables, respectively. The purpose of employing this dataset is not to introduce new data, but to provide a realistic and relevant application that highlights the practical utility of our proposed methods. This data set was also studied by [8]. For detailed information about the dataset, see [37].

Mahto and Tripathi [37] analyzed monthly rainfall data from Long Beach Airport to prepare for potential extreme drought conditions this summer. According to observations, the highest rainfall are seen January, February, March, and December. The data are organized as follows: $X_{1,j}=j=1,2,3,4\;$reflect the monthly rainfall amounts from December 2016 to March 2017, and $Y_{1}$ represents the yearly average rainfall for the 2015–2016 season (defined as the period from July 1 to June 30). Similarly, $X_{2,j}=j=1,2,3,4$ reflects the monthly rainfall from December 2014 to March 2015, whereas $Y_{2}$ the annual average rainfall for the 2013–2014 season. This case originated in the 2005–2006 season. To avoid dependence between the strength and stress variables, data were collected during distinct seasons. A scenario is then constructed assuming that if the rainfall in at least two (or three) out of these four months exceeds the previous year’s annual average rainfall, excessive drought during the summer is unlikely to occur. These scenarios correspond to 2-out-of-4 and 3-out-of-4 systems, respectively.

This data set is as follows:

$$X=\left[\begin{array}{l} 3.59\;\;\;9.33\;\;4.72\;\;0.15\\ 4.41\;\;\;0.87\;\;\;0.24\;\;\;0.49\\ 2.40\;\;\;\;1.04\;\;\;0.30\;\;\;0.85\\ 10.41\;\;1.15\;\;\;1.60\;\;2.67\;\\ 2.61\;\;\;0.17\;\;4.04\;\;\;0.42\\ 0.68\;\;0.20\;\;\;0.49\;\;\;0.03\;\; \end{array}\right]\;\;\;\;\;\;\;\;\;Y=\left[\begin{array}{l} 0.540\\ 0.375\\ 0.631\\ 1.305\\ 0.951\\ 0.718 \end{array}\right]$$

The Kolmogorov–Smirnov (KS) test was used to confirm X and Y IR distribution before we started the estimation. The associated p-value for data set X is 0.1518, and the KS test is 0.6684. For data set Y, it comes out to be 0.4084, with an associated p-value of 0.2641.

To illustrate the proposed methods, we consider the two progressively censored samples with the censoring schemes used by [37].

$$CS1:R=\left[1,0,0\right],\;S=\left[1,0,0,0\right],$$

and

$$CS2:R=\left[0,0,1\right],\;S=\left[0,0,0,1\right].$$

Now, MLE and Bayesian (Lindley and MCMC) of MSSR use Prior-I under SELF, LINEX, and GELF. ACI, BCI, and HPD come with progressively censoring schemes. Table 4 and Table 5 show the outcomes of the real data analysis.

Here, BCI-I, BCI-II, and BCI-III are represented as the BCI intervals of the SELF, LINEX, and GELF loss functions, respectively.

Considering the simulation results presented in Table 4, it can be observed that the LINEX and GELF methods can be preferred for both CS1 and CS2. This is because these methods provide narrower intervals and generally exhibit lower Abias and MSE values in the simulation study. Furthermore, when comparing CS1 and CS2, it is observed that CS1 provides narrower and more precise results in both point and interval estimates. This demonstrates the significant impact of the censoring scheme on estimation accuracy and reliability.

## 6. Conclusions

This study investigated the estimation of MSSR based on the inverse Rayleigh distribution under progressively Type-II censored data. Both MLE and Bayesian methods were employed, where the Bayesian estimators were derived using Lindley’s approximation and the MCMC method. ACIs and BCIs were also constructed. The simulation results demonstrated that Bayesian estimators generally outperform MLEs in terms of Abias and MSE criteria. It is also observed that the MCMC method performs slightly better than the Lindley approximation method under both Prior-I and Prior-II. Additionally, the Bayesian estimators under LINEX and GELF provide better estimates in the sense of having smaller Abias and MSE values. Moreover, it is seen that Prior-II based Bayesian estimators perform better than Prior-I based Bayesian estimators in most cases. A real data application further confirmed the practical utility of the proposed approximations.

Overall, the progressive censoring IR based MSSR model provides a flexible and effective tool for reliability analysis in real-world applications. Its dependence on the IR distribution, however, limits its applicability to specific data types. Future studies should look into applying the suggested techniques to different lifetime distributions in order to increase their flexibility. Furthermore, more comprehensive insights into the robustness of the MSSR estimators could be obtained by investigating the effects of various censoring strategies, such as progressive hybrid censoring. 

Future studies can also investigate the use of sampling techniques like Hamilton Monte Carlo [38] to increase estimation efficiency, particularly in high-dimensional parameter spaces or complex posterior distributions. Moreover, the generality of the model may be demonstrated through real-world applications in technical and biomedical systems. Finally, a development of effective computational algorithms and software tools will enable wider implementation of the suggested techniques.

## Figures and Tables

**Table 1:** Different censoring schemes.

$\left(k,K\right)$		*C.S*	$\left(n,N\right)$		*C.S*
$\;\left(6,16\right)$	$S_{1}$	$\left(4,\;0\times 5\right)$	$\left(12,24\right)$	$R_{1}$	$\left(0\times 11,8\right)$
	$S_{2}$	$\left(0\times 5,4\right)$		$R_{2}$	$\left(8,0\times 11\right)$
	$S_{3}$	$\left(\left(0,1\right)\times 3\right)$		$R_{3}$	$\left(\left(0,1\right)\times 6\right)$
$\left(5,10\right)$	$S_{4}$	$\left(0\times 4,9\right)$	$\left(20,30\right)$	$R_{4}$	$\left(0\times 19,10\right)$
	$S_{5}$	$\left(9,0\times 4\right)$		$R_{5}$	$\left(10,0\times 19\right)$
	$S_{6}$	$\left(2\times 5\right)$		$R_{6}$	$\left(\left(0,1\right)\times 10\right)$

**Table 2:** Abias and MSE values for MLE and Bayesian estimators when $R_{1,3}=0.7895$ and $R_{2,4}=0.6579$.

			$R_{1,3}$				$R_{2,4}$			
			**Prior-I**		**Prior-II**		**Prior-I**		**Prior-II**	
**CS**	**Method**	**LF**	**Abias**	**MSE**	**Abias**	**MSE**	**Abias**	**MSE**	Abias	**MSE**
$\left(R_{1},\;S_{1}\right)$	MLE		0.0023	0.0083	0.0023	0.0083	0.0102	0.0138	0.0102	0.0138
	Lindley	SELF	0.0029	0.0086	0.0026	0.0080	0.0107	0.0154	0.0099	0.0140
		LINEX	0.0022	0.0089	0.0020	0.0081	0.0105	0.0124	0.0096	0.0110
		GELF	0.0027	0.0082	0.0024	0.0079	0.0103	0.0115	0.0094	0.0095
	MCMC	SELF	0.0020	0.0088	0.0021	0.0075	0.0102	0.0096	0.0097	0.0090
		LINEX	0.0018	0.0084	0.0017	0.0081	0.0098	0.0095	0.0090	0.0089
		GELF	0.0015	0.0081	0.0014	0.0077	0.0091	0.0087	0.0084	0.0079
$\left(R_{2,}\;S_{2}\right)$	MLE		0.0027	0.0111	0.0027	0.0111	0.0084	0.0116	0.0047	0.0105
	Lindley	SELF	0.0025	0.0114	0.0020	0.0096	0.0085	0.0105	0.0040	0.0100
		LINEX	0.0018	0.0119	0.0016	0.0099	0.0088	0.0079	0.0030	0.0070
		GELF	0.0024	0.0124	0.0020	0.0094	0.0083	0.0076	0.0039	0.0067
	MCMC	SELF	0.0013	0.0097	0.0011	0.0090	0.0075	0.0069	0.0031	0.0060
		LINEX	0.0014	0.0094	0.0009	0.0084	0.0072	0.0070	0.0027	0.0056
		GELF	0.0016	0.0096	0.0012	0.0087	0.0076	0.0064	0.0030	0.0059
$\left(R_{3,}\;S_{3}\right)$	MLE		0.0030	0.0079	0.0030	0.0079	0.0106	0.0121	0.0106	0.0121
	Lindley	SELF	0.0035	0.0083	0.0028	0.0081	0.0094	0.0117	0.0089	0.0109
		LINEX	0.0038	0.0087	0.0030	0.0084	0.0089	0.0110	0.0080	0.0102
		GELF	0.0033	0.0081	0.0027	0.0077	0.0080	0.0106	0.0074	0.0097
	MCMC	SELF	0.0027	0.0073	0.0024	0.0071	0.0075	0.0102	0.0069	0.0094
		LINEX	0.0024	0.0076	0.0020	0.0069	0.0072	0.0099	0.0066	0.0092
		GELF	0.0025	0.0074	0.0021	0.0064	0.0071	0.0097	0.0064	0.0091
$\left(R_{4,}\;S_{1}\right)$	MLE	MLE	0.0104	0.0121	0.0104	0.0121	0.0123	0.0135	0.0123	0.0135
	Lindley	SELF	0.0073	0.0110	0.0069	0.0096	0.0103	0.0120	0.0095	0.0112
		LINEX	0.0082	0.0113	0.0077	0.0102	0.0108	0.0114	0.0097	0.0104
		GELF	0.0079	0.0118	0.0074	0.0105	0.0106	0.0110	0.0094	0.0102
	MCMC	SELF	0.0107	0.0109	0.0096	0.0100	0.0111	0.0115	0.0102	0.0108
		LINEX	0.0104	0.0101	0.0092	0.0090	0.0104	0.0119	0.0108	0.0103
		GELF	0.0106	0.0096	0.0091	0.0088	0.0106	0.0105	0.0096	0.0096
$\left(R_{5,}\;S_{2}\right)$	MLE		0.0098	0.0138	0.0098	0.0138	0.0109	0.0145	0.0088	0.0137
	Lindley	SELF	0.0059	0.0130	0.0050	0.0120	0.0088	0.0150	0.0079	0.0142
		LINEX	0.0069	0.0121	0.0060	0.0110	0.0076	0.0147	0.0068	0.0135
		GELF	0.0065	0.0128	0.0058	0.0117	0.0084	0.0146	0.0071	0.0129
	MCMC	SELF	0.0059	0.0096	0.0050	0.0091	0.0089	0.0125	0.0081	0.0118
		LINEX	0.0048	0.0099	0.0045	0.0092	0.0077	0.0122	0.0070	0.0114
		GELF	0.0051	0.0094	0.0046	0.0089	0.0071	0.0124	0.0065	0.0113
$\left(R_{6,}\;S_{3}\right)$	MLE		0.0150	0.0119	0.0150	0.0119	0.0162	0.0132	0.0150	0.0126
	Lindley	SELF	0.0106	0.0108	0.0099	0.0100	0.0120	0.0115	0.0108	0.0099
		LINEX	0.0108	0.0103	0.0104	0.0102	0.0114	0.0124	0.0102	0.0110
		GELF	0.0109	0.0109	0.0102	0.0108	0.0115	0.0128	0.0106	0.0107
	MCMC	SELF	0.0083	0.0097	0.0079	0.0097	0.0092	0.0126	0.0087	0.0108
		LINEX	0.0086	0.0094	0.084	0.0094	0.0095	0.0121	0.0084	0.0109
		GELF	0.0082	0.0092	0.0078	0.0096	0.0097	0.0119	0.0091	0.0111
$\left(R_{6,}\;S_{4}\right)$	MLE		0.0121	0.0119	0.0121	0.0119	0.0133	0.0135	0.0120	0.0121
	Lindley	SELF	0.0104	0.0114	0.0099	0.0102	0.0120	0.0127	0.0108	0.0114
		LINEX	0.0102	0.0110	0.0096	0.0100	0.0112	0.0122	0.0106	0.0101
		GELF	0.0108	0.0106	0.0094	0.0102	0.0124	0.0114	0.0114	0.0105
	MCMC	SELF	0.0084	0.0104	0.0079	0.0100	0.0157	0.0151	0.0152	0.0148
		LINEX	0.0082	0.0102	0.0077	0.0096	0.0145	0.0133	0.0141	0.0130
		GELF	0.0088	0.0094	0.0075	0.0092	0.0142	0.0127	0.0139	0.0122
$\left(R_{2,}\;S_{5}\right)$	MLE		0.0087	0.0140	0.0087	0.0140	0.0142	0.0151	0.0142	0.0151
	Lindley	SELF	0.0084	0.0136	0.0080	0.0132	0.0135	0.0146	0.0132	0.0143
		LINEX	0.0073	0.0132	0.0069	0.0128	0.0124	0.0157	0.0120	0.0151
		GELF	0.0077	0.0124	0.0074	0.0119	0.0117	0.0157	0.0112	0.0153
	MCMC	SELF	0.0069	0.0120	0.0066	0.0116	0.0093	0.0152	0.0090	0.0148
		LINEX	0.0060	0.0116	0.0056	0.0110	0.0088	0.0113	0.0082	0.0111
		GELF	0.0062	0.0113	0.0059	0.0109	0.0080	0.0148	0.0077	0.0141
$\left(R_{3,}\;S_{2}\right)$	MLE		0.0067	0.0136	0.0067	0.0136	0.0096	0.0142	0.0094	0.0139
	Lindley	SELF	0.0036	0.0121	0.0029	0.0118	0.0094	0.0145	0.0091	0.0140
		LINEX	0.0028	0.0127	0.0024	0.0120	0.0092	0.0123	0.0089	0.0119
		GELF	0.0022	0.0123	0.018	0.0111	0.0079	0.0119	0.0072	0.0111
	MCMC	SELF	0.0026	0.0108	0.0020	0.0097	0.0060	0.0095	0.0058	0.0091
		LINEX	0.0025	0.0106	0.0018	0.0094	0.0051	0.0119	0.0049	0.0102
		GELF	0.0021	0.0099	0.0016	0.0092	0.0048	0.0110	0.0044	0.0099
$\left(R_{4,}\;S_{4}\right)$	MLE		0.0107	0.0137	0.0107	0.0137	0.0125	0.0158	0.0121	0.0152
	Lindley	SELF	0.0104	0.0132	0.0096	0.0120	0.0118	0.0156	0.0111	0.0150
		LINEX	0.0098	0.0134	0.0090	0.0125	0.0109	0.0163	0.0102	0.0158
		GELF	0.0085	0.0140	0.0079	0.0130	0.0102	0.0161	0.0099	0.0156
	MCMC	SELF	0.0100	0.0090	0.0092	0.0080	0.0110	0.0129	0.0105	0.0109
		LINEX	0.0102	0.0096	0.0090	0.0090	0.0105	0.0121	0.0101	0.0114
		GELF	0.0104	0.0094	0.0099	0.0088	0.0109	0.0123	0.0102	0.0118
$\left(R_{5,}\;S_{5}\right)$	MLE		0.0157	0.0192	0.0157	0.0192	0.0173	0.0175	0.0173	0.0175
	Lindley	SELF	0.0129	0.0193	0.0120	0.0187	0.0136	0.0156	0.0132	0.0150
		LINEX	0.0120	0.0172	0.0115	0.0167	0.0130	0.0153	0.0126	0.0148
		GELF	0.0127	0.0181	0.0118	0.0170	0.0124	0.0144	0.0139	0.0140
	MCMC	SELF	0.0119	0.0187	0.0110	0.0168	0.0115	0.0125	0.0110	0.0119
		LINEX	0.0120	0.0180	0.0109	0.0167	0.0104	0.0121	0.0100	0.0118
		GELF	0.0122	0.0176	0.0111	0.0160	0.0109	0.0119	0.0103	0.0112
$\left(R_{6,}\;S_{6}\right)$	MLE		0.0127	0.0165	0.0127	0.0188	0.0145	0.0188	0.0141	0.0180
	Lindley	SELF	0.0105	0.0162	0.0099	0.0165	0.0120	0.0180	0.0115	0.0175
		LINEX	0.0116	0.0164	0.0108	0.0156	0.0123	0.0178	0.0120	0.0170
		GELF	0.0112	0.0166	0.0104	0.0128	0.0118	0.0147	0.0111	0.0141
	MCMC	SELF	0.0104	0.0123	0.0099	0.0126	0.0107	0.0140	0.0102	0.0132
		LINEX	0.0105	0.0120	0.0099	0.0122	0.0111	0.0136	0.0107	0.0130
		GELF	0.0107	0.0119	0.0094	0.0118	0.0113	0.0129	0.0108	0.0121

**Table 3:** ACI, CP and BCI estimators for $R_{1,3}$ and $R_{2,4}$.

		$R_{1,3}$		$R_{2,4}$	
**CS**	**Method**	**ACI/BCI**	**CP**	**ACI/BCI**	**CP**
$\left(R_{1},\;S_{1}\right)$	MLE	0.1488	0.9221	0.1494	0.9140
	Prior-I	0.1325	0.9285	0.1461	0.9187
	Prior-II	0.1302	0.9302	0.1402	0.9248
$\left(R_{2},\;S_{2}\right)$	MLE	0.1561	0.9240	0.1466	0.9231
	Prior-I	0.1423	0.9360	0.1440	0.9260
	Prior-II	0.1392	0.9389	0.1435	0.9288
$\left(R_{3},\;S_{3}\right)$	MLE	0.1502	0.9185	0.1604	0.9225
	Prior-I	0.1441	0.9204	0.1588	0.9302
	Prior-II	0.1402	0.9225	0.1568	0.9356
$\left(R_{4},\;S_{1}\right)$	MLE	0.1615	0.9360	0.1702	0.9280
	Prior-I	0.1540	0.9402	0.1680	0.9310
	Prior-II	0.1488	0.9456	0.1656	0.9338
$\left(R_{5},\;S_{2}\right)$	MLE	0.1593	0.9405	0.1658	0.9296
	Prior-I	0.1562	0.9554	0.1589	0.9355
	Prior-II	0.1485	0.9501	0.1545	0.9390
$\left(R_{6},\;S_{3}\right)$	MLE	0.1586	0.9340	0.1674	0.9168
	Prior-I	0.1462	0.9412	0.1580	0.9225
	Prior-II	0.1405	0.9456	0.1503	0.9298
$\left(R_{1},\;S_{4}\right)$	MLE	0.1669	0.9460	0.1788	0.9302
	Prior-I	0.1527	0.9494	0.1704	0.9374
	Prior-II	0.1486	0.9402	0.1688	0.9413
$\left(R_{2},S_{5}\right)$	MLE	0.1772	0.9420	0.1844	0.9325
	Prior-I	0.1688	0.9502	0.1814	0.9390
	Prior-II	0.1602	0.9556	0.1758	0.9457
$\left(R_{3},S_{6}\right)$	MLE	0.1631	0.9356	0.1717	0.9248
	Prior-I	0.1525	0.9440	0.1650	0.9335
	Prior-II	0.1517	0.9502	0.1602	0.9441
$\left(R_{4},S_{4}\right)$	MLE	0.1600	0.9340	0.1808	0.9185
	Prior-I	0.1584	0.9419	0.1758	0.9225
	Prior-II	0.1498	0.9496	0.1714	0.9302
$\left(R_{5},S_{5}\right)$	MLE	0.1671	0.9480	0.1813	0.9265
	Prior-I	0.1637	0.9502	0.1788	0.9224
	Prior-II	0.1613	0.9592	0.1712	0.9393
$\left(R_{6},S_{6}\right)$	MLE	0.1713	0.9440	0.1878	0.9224
	Prior-I	0.1690	0.9497	0.1817	0.9302
	Prior-II	0.1602	0.9541	0.1756	0.9425

**Table 4:** The MLE and Bayesian estimations of the $R_{s,k}$ in the case of monthly rainfall data.

			Lindley			MCMC		
		MLE	SELF	LINEX	GELF	SELF	LINEX	GELF
CS1	$R_{13}$	0.5257	0.5251	0.52454	0.5253	0.5160	0.5159	0.5161
	$R_{24}$	0.3135	0.3133	0.3130	0.3133	0.3032	0.3029	0.3034
CS2	$R_{13}$	0.5749	0.5632	0.5625	0.5630	0.5542	0.5540	0.5544
	$R_{24}$	0.3695	0.3588	0.3892	0.3597	0.3458	0.3451	0.3453

**Table 5:** The interval estimations of the $R_{s,k}$ in the case of monthly rainfall data.

		ACI	BCI-I	BCI-II	BCI-III
CS1	$R_{13}$	(0.3744 0.6798)	(0.3733 0.6790)	(0.3740 0.6792)	(0.3738 0.6790)
	$R_{24}$	(0.3845 0.6905)	(0.3843 0.6898)	(0.3839 0.6890)	(0.3836 0.6887)
CS2	$R_{13}$	(0.1652 0.5012)	(0.1693 0.5000)	(0.1690 0.4998)	(0.1688 0.4987)
	$R_{24}$	(0.1748 0.5162)	(0.1741 0.5127)	(0.1737 0.5120)	(0.1735 0.5118)

## Data Availability

The original contributions presented in this study are included in the article. Further inquiries can be directed to the corresponding author.
